# Global research trends on epigenetics and neuropathic pain: A bibliometric analysis

**DOI:** 10.3389/fnmol.2023.1145393

**Published:** 2023-04-19

**Authors:** Chenchen Zhu, Weiquan Zhong, Chan Gong, Binglin Chen, Jiabao Guo

**Affiliations:** The Second School of Clinical Medical College, Xuzhou Medical University, Xuzhou, Jiangsu, China

**Keywords:** neuropathic pain, epigenetics, bibliometrics, CiteSpace, Web of Science

## Abstract

**Objective:**

Neuropathic pain (NP) is a common disease that manifests with pathological changes in the somatosensory system. In recent years, the interactions of NP with the epigenetic mechanism have been increasingly elucidated. However, only a few studies have used bibliometric tools to systematically analyze knowledge in this field. The objective of this study is to visually analyze the trends, hotspots, and frontiers in epigenetics and NP research by using a bibliometric method.

**Methods:**

Studies related to epigenetics and NP were searched from the Science Citation Index-Expanded of the Web of Science Core Collection database. Search time is from inception to November 30, 2022. No restrictions were placed on language. Only articles and reviews were included as document types. Data on institutions, countries, authors, journal distribution, and keywords were imported into CiteSpace software for visual analysis.

**Results:**

A total of 867 publications met the inclusion criteria, which spanned the period from 2000 to 2022. Over the years, the number of publications and the frequency of citations exhibited a clear upward trend in general, reaching a peak in 2021. The major contributing countries in terms of the number of publications were China, the United States, and Japan. The top three institutions were Rutgers State University, Xuzhou Medical University, and Nanjing Medical University. Molecular Pain, Pain, and Journal of Neuroinflammation contributed significantly to the volume of issues. Among the top 10 authors in terms of the number of publications, Tao Yuan-Xiang contributed 30 entries, followed by Zhang Yi with 24 and Wu Shao-Gen with 20. On the basis of the burst and clusters of keywords, “DNA methylation,” “Circular RNA,” “acetylation,” “long non-coding RNA,” and “microglia” are global hotspots in the field.

**Conclusion:**

The bibliometric analysis indicates that the number of publications related to epigenetics and NP is exhibiting a rapid increase. Keyword analysis shows that “DNA methylation,” “Circular RNA,” “acetylation,” “long non-coding RNA” and “microglia” are the most interesting terms for researchers in the field. More rigorous clinical trials and additional studies that explore relevant mechanisms are required in the future.

## 1. Introduction

Epigenetic modifications, including DNA methylation, histone modifications, non-coding RNA, and RNA modification, modulate gene expression without altering the underlying DNA sequence (Portela and Esteller, 2010). Therefore, epigenetic mechanisms might interfere with protein expression and function but may not modify the code of the gene. As we know, most chronic conditions are caused by complex interactions between genes and the environment (e.g., exposure to toxic chemical elements, nutrition, physical activity, and stress), while epigenetics constructs the link between the environment and gene function (Chappell et al., 2016; Cavalli and Heard, 2019; Ferioli et al., 2019; Park et al., 2019; Polli et al., 2019). Neuropathic pain (NP) is typically a chronic condition that manifests itself with pathological changes in the somatosensory system (Jensen et al., 2011). Recent studies have demonstrated that the relevant underlying mechanisms of NP are related to epigenetic modifications (Frances et al., 2022; Jiang et al., 2022). The epigenetic regulator plays a crucial role in the maintenance and development of NP by activating and suppressing various gene expressions, such as neuroinflammatory factors, neurotransmitters, receptors, and ion channels (Franco-Enzastiga et al., 2021; Borgonetti et al., 2022; Sun et al., 2022; Zhang J. et al., 2022). Given that the publication of epigenetics and NP is rapidly evolving, understanding the research progress, trends, and hotspots in this field is necessary for relevant researchers. However, a systematic and visualized analysis that shows the global trends on epigenetic research in NP is still lacking.

A bibliometric analysis is a scientific method for evaluating published scholarly literature, searching for data correlations, and predicting future developments for a particular research field (Hicks et al., 2015). Furthermore, the software CiteSpace has been widely used for quantitative analysis and mapping visualization (Chen, 2006). Specifically, the data on institutions, journals, countries, authors, and keywords can be analyzed. A few reviews have focused on knowledge about epigenetic modifications in the development and maintenance of NP. However, a bibliometric analysis remains lacking.

In the current work, a bibliometric study was conducted to analyze publications related to NP in the epigenetic field retrieved from the Web of Science (WoS) database from 2000 to 2022. Our study aims to summarize research progress and trends related to NP in apparent modifications to help researchers quickly understand the field and guide future research directions.

## 2. Analytic methods

### 2.1. Data sources

We conducted a search for studies on epigenetics and NP from the Science Citation Index-Expanded (SCIE) of the WoS Core Collection database. The investigation was performed from inception until November 30, 2022. No restrictions were imposed on language. The search strategy is provided in Table 1. A total of 867 matching records were finally included, and the data were exported in plain text format of the entire record, called “download_XXX.”

**Table 1:** Search strategy of our study.

Set	Search query
#1	TS = (“Neuropathic Pain” OR “Trigeminal Neuralgia” OR “Postherpetic Neuralgia” OR “Pain After Peripheral Nerve Injury” OR “Painful Polyneuropathy” OR “Painful Radiculopathy” OR “Painful Diabetic Neuropathy” OR “Diabetic Painful Neuropathy” OR “Chemotherapy Induced Peripheral Neuropathy” OR “Chronic Inflammatory Demyelinating Polyneuropathy” OR “Neuropathic Cancer Pain” OR “Spinal Cord Injury Pain” OR “Central Post-Stroke Pain”)
#2	TS = (epigenetic* OR epigenomic* OR “DNA methylation” OR methyltransferase* OR demethylase* OR Hypermethylation OR Hypomethylation OR “CpG island” OR “histone modification” OR “histone methylation” OR “histone phosphorylation” OR “histone deacetylase*” OR “histone acetyltransferase*” OR “chromatin remodeling” OR “non-coding RNA*” OR MicroRNA* OR “micro RNAs” OR “micro RNA” OR micro-RNAs OR micro-RNA OR lncRNA* OR “long ncRNA*” OR “long noncoding RNA*” OR “Long Non-Coding RNA*” OR circRNA* OR “Circular RNA*” OR “circ-RNA” OR “RNA modification” OR N6-methyladenosine OR 5-methylcytosine)

### 2.2. Data analysis

**Figure 1:**
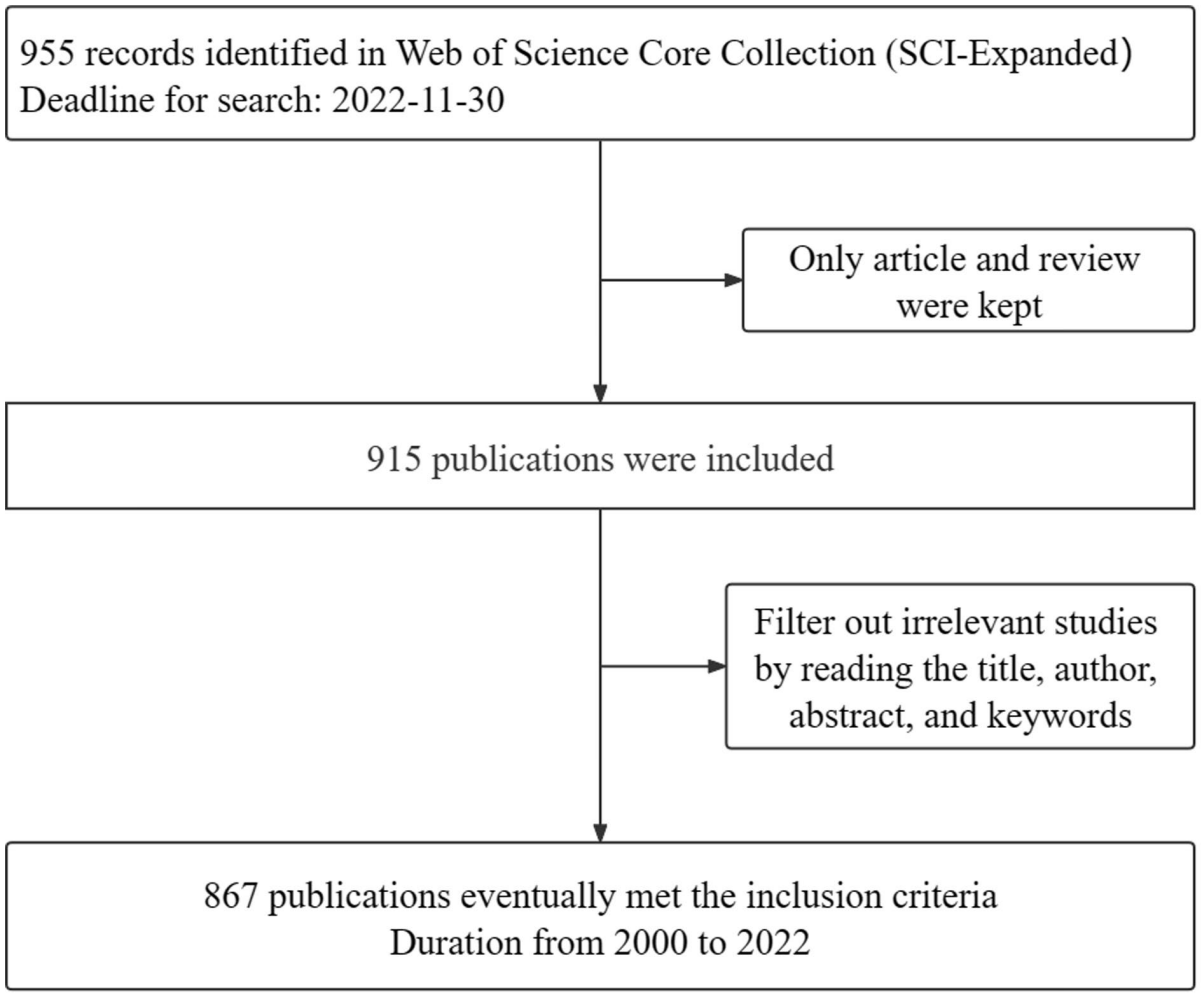
Flowchart of the publication screening process.

CiteSpace is a well-known literature analysis software that uses the interrelationships between words (e.g., authors, journals, and keywords, etc.) in academic literature to visualize scientific research trends. All downloaded plain text formats were imported into CiteSpace software (6.1.R2). The first step was to remove duplicates, and then save the deduplication data in the output and data folders. Ultimately, 867 eligible documents were accessed, spanning from 2000 to 2022. The process flow is shown in Figure 1. The parameters of CiteSpace were set as follows. “Time Slicing” was set from 2000.01 to 2022.12. Years per slice was set to 1. The rest was set to the specific value, and the cutting method can be set as necessary in accordance with the selected nodes, such that the result would be the most stable, transparent, and intuitive. The corresponding visual map was drawn and interpreted.

## 3. Results

### 3.1. Publication outputs

**Figure 2:**
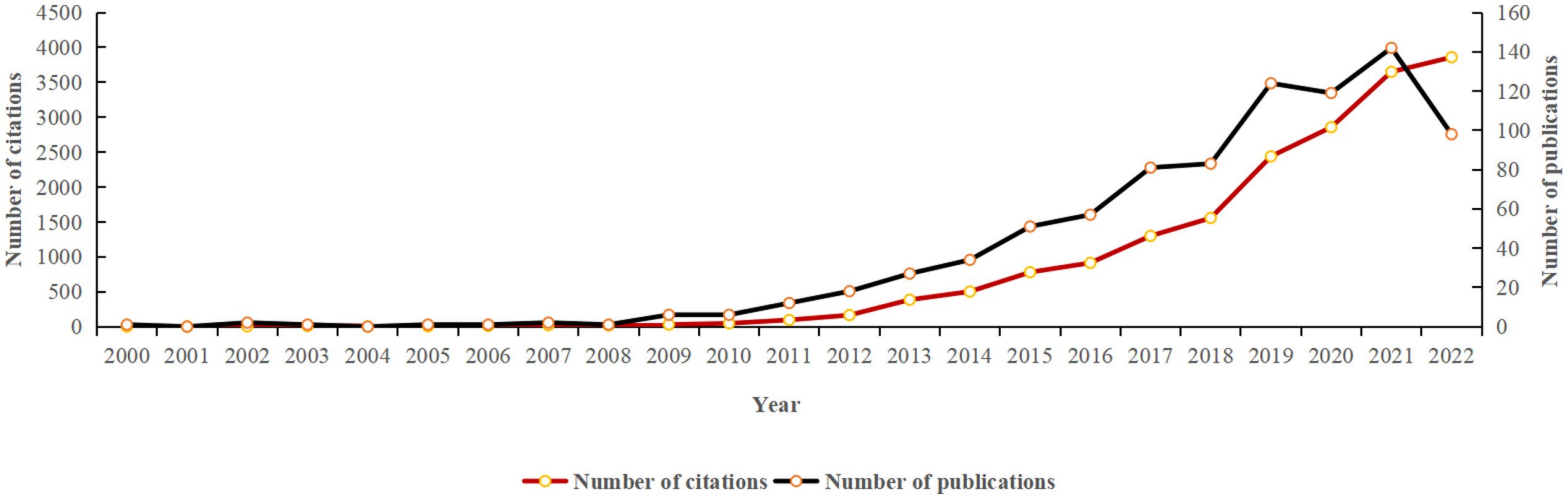
The annual number of publications and citations on epigenetics and NP from 2000 to 2022.

A total of 867 data items, including 720 articles and 147 reviews, were analyzed in the current study. As shown in Figure 2, the overall number of publications and citations per year exhibits an upward trend. Although the number of articles published from 2019 to 2020 presented a short-term decline, the number of articles published in the following year reached a peak. In 2021, the number of publications stood at 142, and the citations amounted to 3,651.

### 3.2. Analysis of countries/regions and institutions

**Figure 3:**
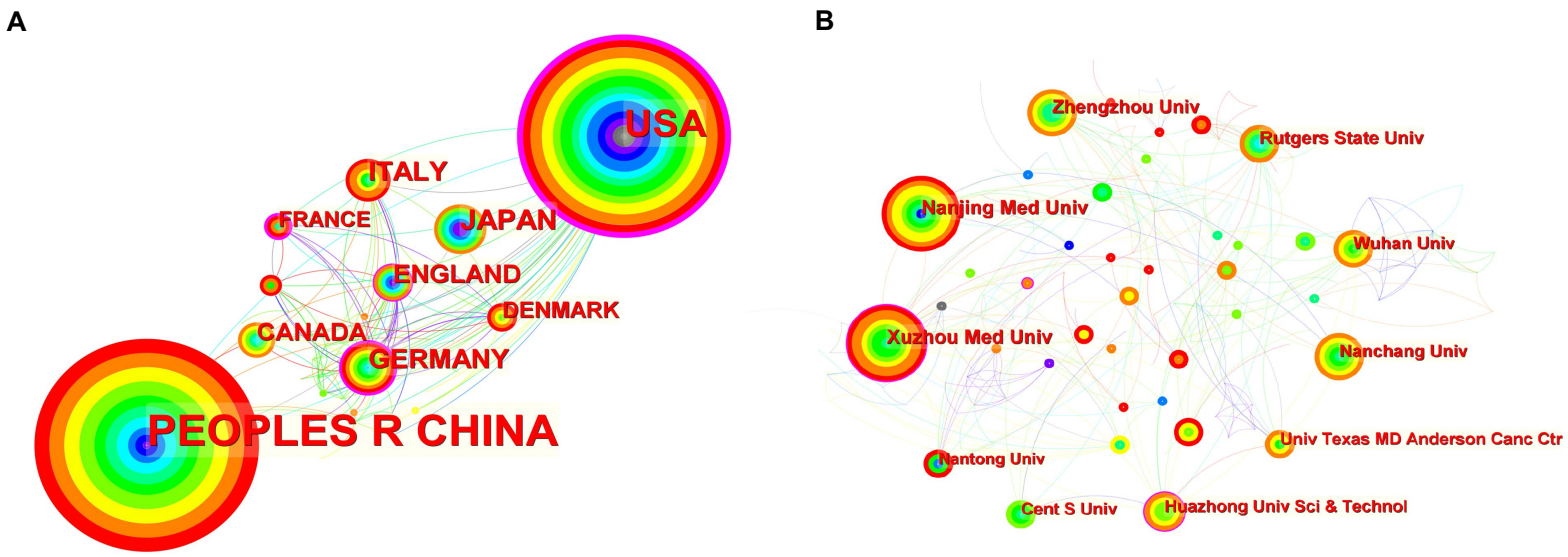
**(A)** Co-occurrence relationship of countries. **(B)** Partnership between the institutions. The nodes in the map represent co-countries/co-institutions, and the lines between the nodes represent co-citation relationships. The warm and cold colors represent the distance of time of their first co-occurrence, the warmer the color, the more recent the time. The purple ring represents centrality.

Figure 3A shows the co-occurrence relationship among countries. Table 2 lists the detailed information of the top 10 countries in accordance with the number of publications. The Country’s frequent count is based on the affiliation of all authors, and the top three countries in issuance are China, the United States, and Japan. China has the most significant number of publications with 481 pieces, accounting for 55.48% of all the papers enrolled in this study, and the total number of citations and the H-index are both the highest. The country with the highest number of citations per paper is Japan. Centrality indicates that one node builds a bridge between two unrelated nodes, and the higher the centrality, the more influential the node is in the structure. In CiteSpace, a node with a centrality of >0.1 is called a critical node. The top three countries are the United States (0.62), Germany (0.42), and France (0.23). Figure 3B and Table 3 illustrate the partnership and details among institutions. The top three institutions are Rutgers State University (*n* = 87, centrality = 0.08), Xuzhou Medical University (*n* = 41, centrality = 0.16), and Nanjing Medical University (*n* = 37, centrality = 0.05).

**Table 2:** Analysis of the top 10 countries.

Country/Region	Publications	Centrality	Citations	Citations per paper	H-index
China	481	0.13	8,102	16.84	44
United States	206	0.62	6,112	29.67	41
Japan	48	0.00	1889	39.35	27
Germany	39	0.42	1,227	28.90	17
Italy	37	0.02	787	21.27	16
England	31	0.10	1,145	36.94	14
Canada	27	0.00	786	29.11	14
Denmark	19	0.02	710	37.37	9
France	18	0.23	409	22.72	9
South Korea	15	0.00	273	18.20	8

**Table 3:** Analysis of the top 10 institutions.

Rank	Institution	Publications	Rank	Institution	Centrality
1	Rutgers State University	87	1	Xuzhou Medical University	0.16
2	Xuzhou Medical University	41	2	Huazhong University Science and Technology	0.13
3	Nanjing Medical University	37	3	Harvard Medical School	0.13
4	Zhengzhou University	31	4	McGill University	0.09
5	University of Texas System	28	5	Jilin University	0.09
6	Central South University	26	6	University Minnesota	0.09
7	Wuhan University	25	7	Rutgers State University	0.08
8	Nanchang University	23	8	China Medical University	0.07
9	University of London	23	9	UTMD Anderson Cancer Center	0.05
10	UTMD Anderson Cancer Center	23	10	Nantong University	0.05

### 3.3. Analysis of the top 10 authors

Among the top 10 authors in terms of the number of publications, Tao Yuan-Xiang contributed 30 entries, followed by Zhang Yi with 24 and Wu Shao-Gen with 20 (Table 4). Tao’s articles ranked first in terms of the total number of citations and the H-index.

**Table 4:** Top 10 most productive authors.

Author	Publications	Citations	Citations per item	H-index
Tao Yuan-Xiang	30	1,044	34.80	17
Zhang Yi	24	358	14.92	12
Wu Shao-Gen	20	614	30.70	15
Bekker Alex	19	632	33.26	13
Guo Qu-Lian	18	452	25.11	11
Zhang Juan	18	315	17.50	12
Wang Jian	17	232	13.65	10
Wang Yun	15	420	28.00	10
Liang Ling Li	14	756	54.00	11
Mo Kai	14	511	36.50	13

### 3.4. Analysis of cited journals

The top 10 journals with the highest total counts are shown in Table 5. The top 10 journals published 214 papers, accounting for 24.68% of the total. The impact factor (IF) of these journals in 2021 ranged from 2.832 to 9.587. Among them, Molecular Pain (*n* = 46, IF 2021, 3.370), Pain (*n* = 39, IF 2021, 7.926), and Journal of Neuroinflammation (*n* = 20, IF 2021, 9.587) contributed significantly to the volume of issues, with an average of 35 articles per journal. Among the 10 journals, 40% were Q1, representing the top 25% of IF distribution.

**Table 5:** Top 10 most productive journals.

Journal	Counts	IF (2021)
Molecular Pain	46	3.370
Pain	39	7.926
Journal of Neuroinflammation	20	9.587
Journal of Pain Research	19	2.832
Neurochemical Research	17	4.414
Frontiers in Molecular Neuroscience	15	5.512
Journal of Cellular Biochemistry	15	4.480
Journal of Neuroscience	15	6.709
International Journal of Molecular Sciences	14	5.314
Neuroscience	14	3.708

### 3.5. Analysis of keywords

**Figure 4:**
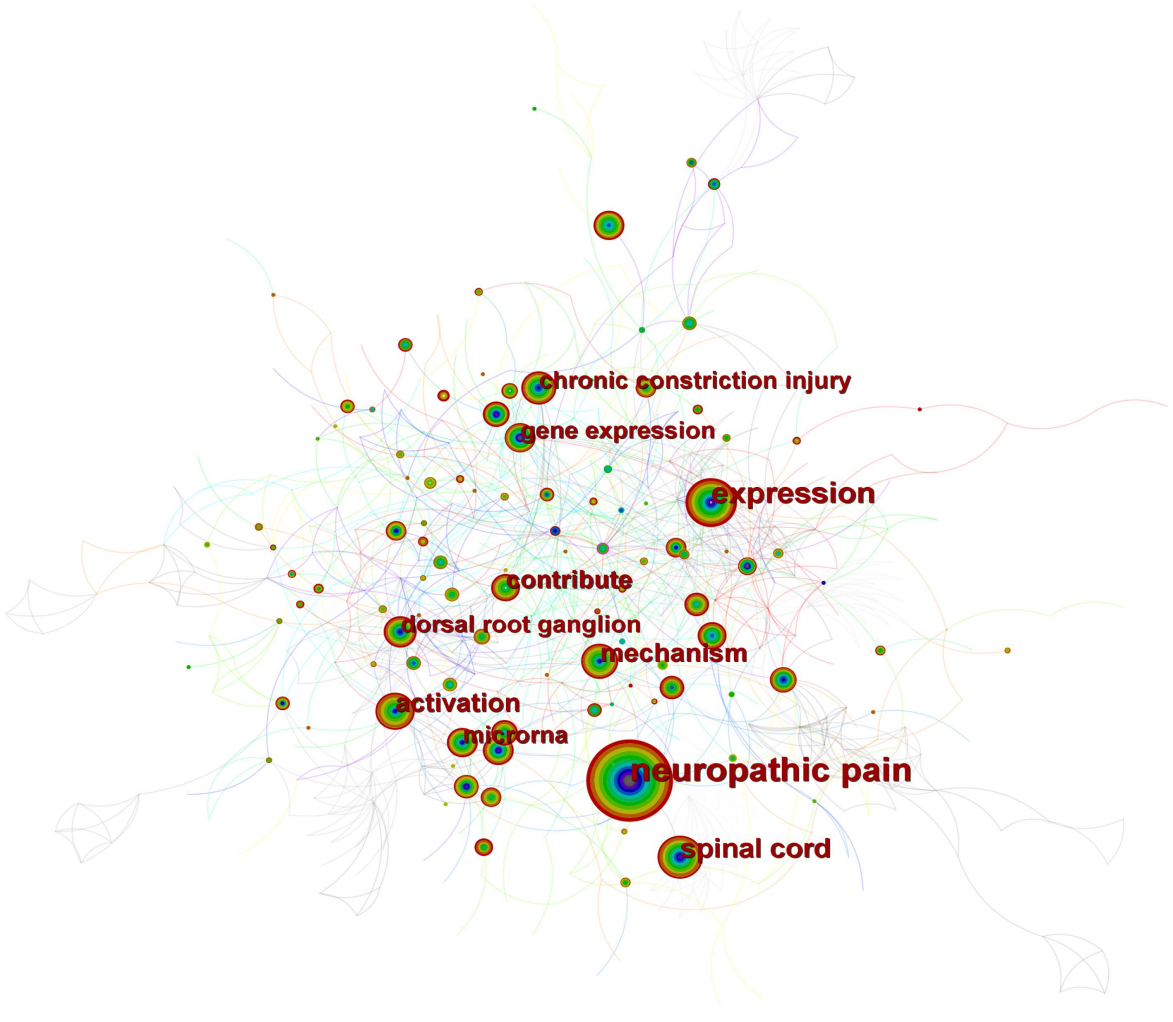
A map of keywords. The circle size and the link illustrated the frequency and relevance of keywords. The thickness of a color wheel is proportional to the number of occurrences of the keyword at that period.

**Figure 5:**
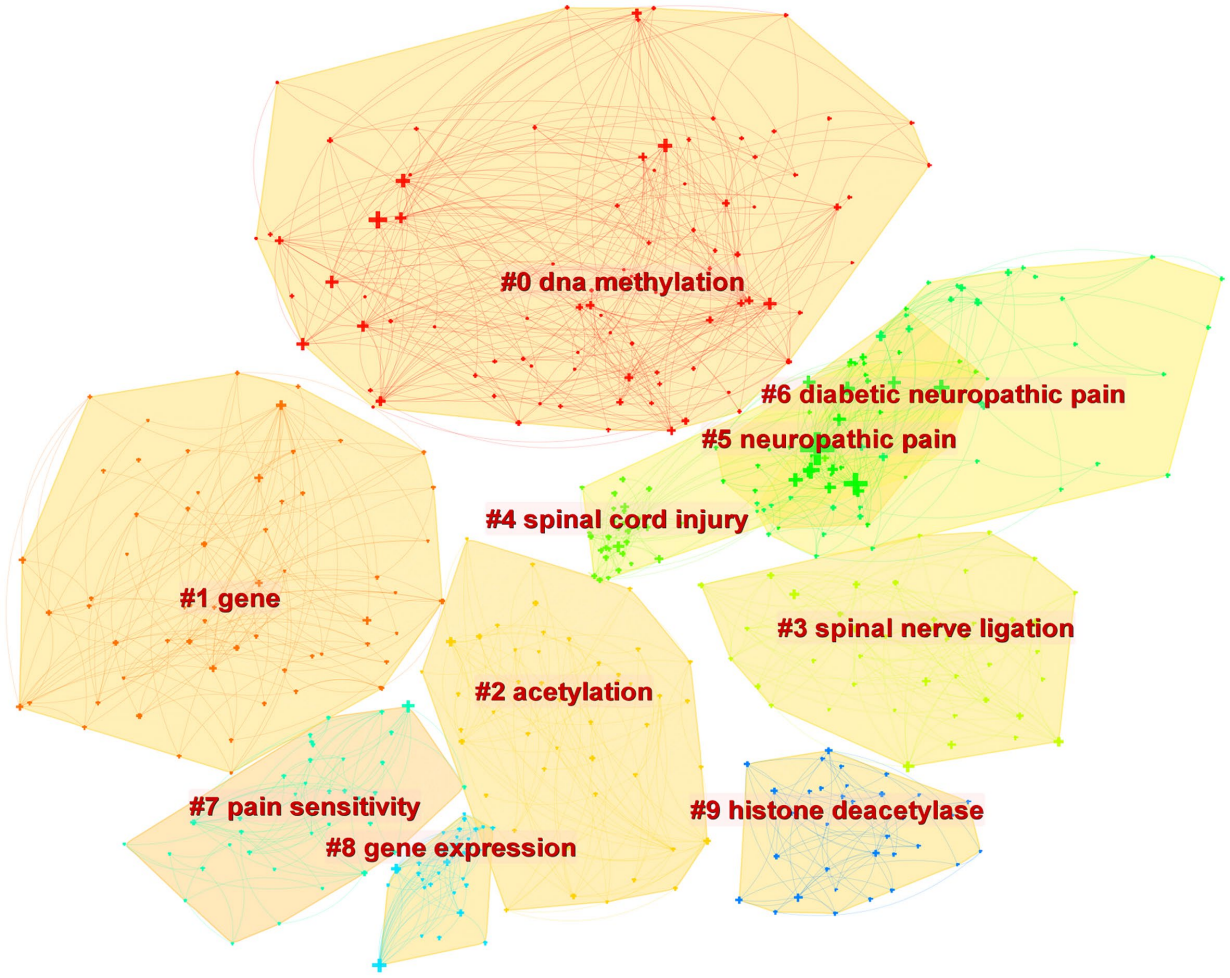
The cluster diagram for the keywords.

**Figure 6:**
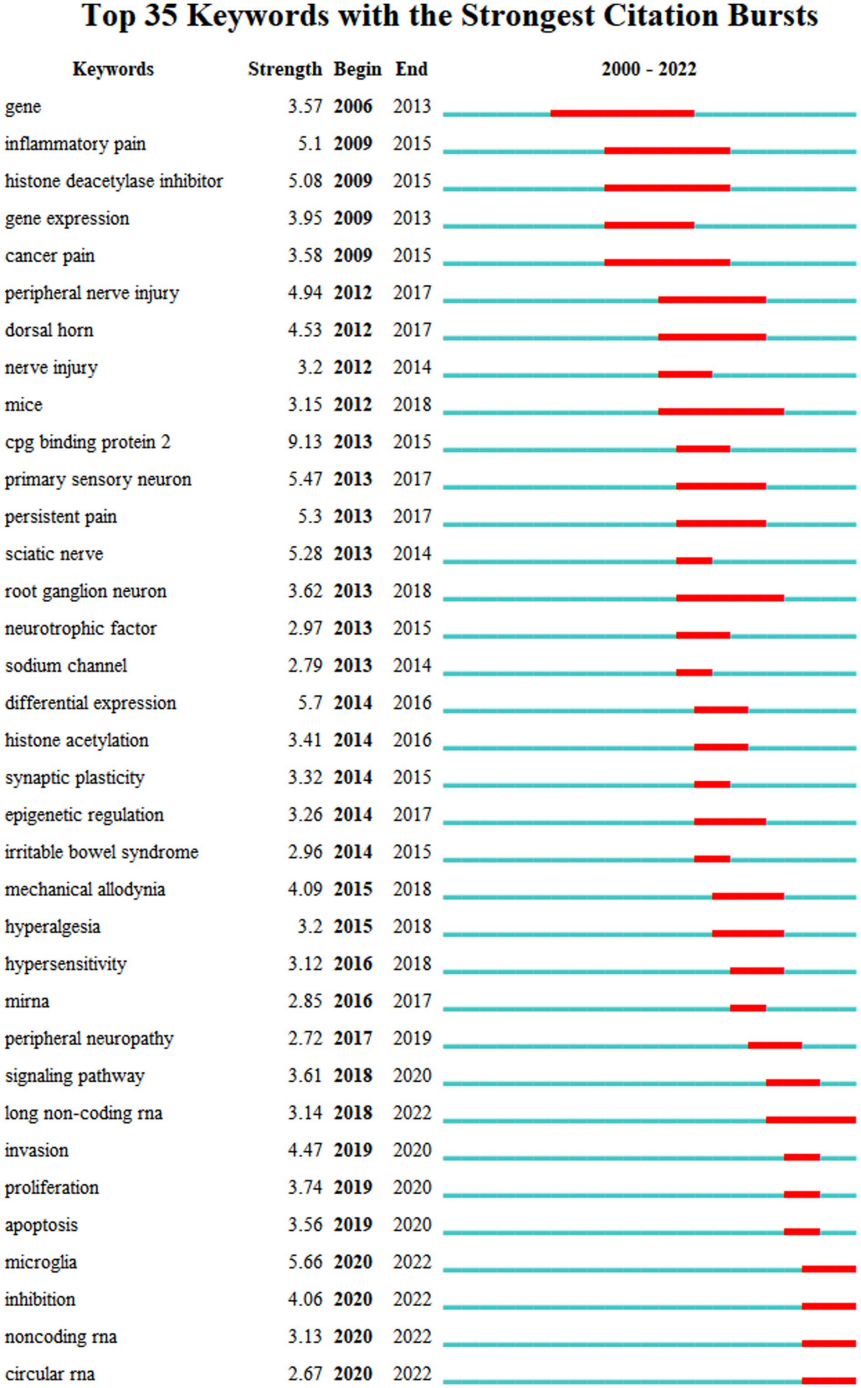
The top 35 keywords with the strongest citation bursts of publications. The “Begin” and “End” indicate the start and termination years of the keyword as a frontier. The “Strength” indicates the intensity of the emergence. The red line represents the specific period when the keyword became a research hotspot, and the light blue means that the node has not yet appeared.

Figure 4 depicts the co-occurrence structure of keywords. The five major keywords that appear most frequently are “neuropathic pain” (*n* = 601), “expression” (*n* = 268), “spinal cord” (*n* = 152), “mechanism” (*n* = 128), and “activation” (*n* = 114). The modularity value (*Q* value) and the average silhouette value (*S* value) are two important parameters for evaluating the effectiveness of mapping (Figure 5). A *Q* value >0.3 and an *S* value >0.7 indicate significant clustering (Zheng et al., 2021). For the current study, the *Q*-value was 0.4331 and the *S*-value was 0.7293, indicating that these clusters were effective and exhibited good homogeneity. The front five clusters were #0 DNA methylation, #1 gene, #2 acetylation, #3 spinal nerve ligation, and #4 spinal cord injury. Figure 6 shows 35 keywords with a sudden increase in frequency and provides a basis for predicting the research focus of this field, which are analyzed in two dimensions: burst value and burst period. Its principle for measuring burst intensity and persistence is the algorithm for detecting frequency bursts suggested by Kleinberg (2002). The approach is based on modeling the stream by using an infinite-state automaton, in which bursts appear naturally as state transitions (Kleinberg, 2002). The top five keywords in terms of burst strength are “CPG binding protein 2” (9.13), “differential expression” (5.7), “microglia” (5.66), “primary sensory neuron” (5.47), and “persistent pain” (5.30). Based on the duration of the burst, the keywords with the longest outbreak duration were “gene,” followed by “inflammatory pain” and “histone deacetylase inhibitor.” “long non-coding RNA,” “microglia,” “inhibition,” “noncoding RNA” and “circular RNA” are keywords that continue to bursting until 2022, which shows that they are recent hotspots.

## 4. Discussions

### 4.1. General trends on epigenetics and NP

In the studied 22 years, the total number of publications grew steadily, reaching a peak in 2021. We segmented the period of the study into two phases, referring to the period from 2000 to 2012 as the initial stage, and from 2013 to the present as the development stage. The rate of ascendancy from 2000 to 2012 was relatively flat, with the number of papers not exceeding 18 and the citations not higher than 164. But from 2013 to the present, the number of published articles has increased by leaps and bounds. Among the total of 867 literatures, only five clinical studies are identified (Huang et al., 2017; Mathias et al., 2017; Li et al., 2020; Fernandez-de-las-Penas et al., 2022; Stenz et al., 2022). Most of them focus on peripheral NP to search for potential epigenetic markers. For example, Mathias et al. showed that in white blood cells, miR-21 and miR-146a expression were increased compared to healthy controls, while miR155 expression was lower. In the peroneal nerve, miR-21 was increased. In painful neuropathy, miR-146a and miR-155 expression were reduced compared to thighs in lower leg skin biopsies. Therefore, peripheral neuropathy is related to abnormal miRNA expression in leukocytes, gastrocnemius nerve and skin. However, the samples included in these studies were small, and these findings will require more adequate studies in the future.

Among the top 10 authoritative authors in terms of the number of publications, a highly cited article by Tao Yuan-Xiang, who ranked first, caught our attention. The most frequently cited paper written by Tao Yuan-Xiang is “A long noncoding RNA contributes to NP by silencing Kcna2 in primary afferent neurons” (232 times) (Zhao et al., 2013). This study proposes that endogenous Kcna2 antisense RNA could be a therapeutic target for the treatment of NP, and the significance of a long non-coding RNA (lncRNA) for the analysis of NP was highlighted. Among the top three authors analyzed above, Tao Yuan-Xiang works for Rutgers State University Medical Center. However, the centrality of cooperation among the authors in the studies is generally less than 0.1, indicating that the cooperation among researchers is not so close, and there are certain limitations.

Countries such as China and the United States have made important contributions to the publication of the articles. The total number of articles cited and the H-index of China are higher than those of the United States, while its citations per paper are the lowest. The United States has cooperated with many countries in this field and has a high centrality (centrality = 0.62), while China (centrality = 0.13) is relatively lacking in international cooperation, probably due to the fact that the United States, as a developed country, has numerous research institutions and extensive collaborations worldwide, and has invested heavily in research in the fields of epigenetics and NP. The top three institutions in terms of the number of published papers are all universities. However, Figure 3B shows that the locational relationships among institutions are relatively fragmented, indicating that academic cooperation and exchange among institutions are insufficient. Therefore, countries and institutions should continue to strengthen communication and collaboration to conduct multicenter and large-sample studies.

The top 10 journals accounted for 24.68% (214 publications) of the number of all published papers. Among the top 10 journals, Molecular Pain, Pain, and Journal of Neuroinflammation have the largest number of publications. Journal of Neuroinflammation has the highest impact factor (IF 2021, 9.587), and 40% of the top 10 journals belong to Q1. This finding indicates that many high-quality journals participate in the field, and thus, it has promising research prospects.

### 4.2. Research hotspots and frontiers

#### 4.2.1. DNA methylation

As shown in Figure 5, DNA methylation emerged as the largest cluster. In humans, DNA methylation is triggered by DNA methyltransferases (DNMTs), which primarily add methyl groups from *S*-adenosyl-l-methionine to the carbon-5 position of cytosine bases [5-methylcytosine (5mC)] located mainly in cytosine-phosphate-guanosine (CpG) islands (Cheng and Roberts, 2001; Holliday, 2005). The promoters of most genes, particularly housekeeping genes, are positioned on CpG islands, which are highly conserved, suggesting that these regions have important functions (Bird et al., 1985). Methylation of CpG islands breaks transcription factor (TF) binding and recruits DNMT-mediated Methyl-CpG-Binding Domain (MBD) Proteins to silence gene expression. Methyl-CpG-binding protein 2 (MeCP2) is a family member of MBD that acts mainly as a transcriptional repressor. It has been shown that The knockout of MeCP2 restores Mu opioid receptor (MOR) expression in damaged dorsal root ganglion (DRG) and enhances the analgesic effect of morphine, suggesting that the downregulation of MOR in DRG is closely associated with increased MeCP2 expression (Zhang et al., 2014; Sun et al., 2021). This unique gene expression raises the possibility that DNA methylation is related to the pathological mechanisms of NP and that further studies on the role of MeCP2 in NP are necessary.

#### 4.2.2. Circular RNA

The keyword “Circular RNA” exploded in the last 3 years in accordance with the burst diagram of the keywords (Figure 6); it represented one of the latest hotspots in this field. Circular RNA (circRNA) is a novel type of lncRNA characterized by a covalently closed loop structure at their 3′ and 5′ ends, and it is highly stable and evolutionarily conserved (Sanger et al., 1976; Memczak et al., 2013). With the rapid development of high-throughput sequencing and bioinformatics algorithms in recent years, the role of circRNA in NP has been extensively studied (Mao et al., 2019; Pan et al., 2019; Zhang et al., 2020). Some dysregulated circRNAs (e.g., circ-Filip1l, circHIPK3, ciRS-7, circRNA.2837, circ-Ankib1 and circAnks1a) have been detected and validated *via* qRT-PCR (Zhou et al., 2018; Mao et al., 2019; Cai et al., 2020; Yin et al., 2022). For example, Cai et al. (2020) found that ciRS-7 can regulate autophagy and inflammation in rats with chronic constriction injury (CCI) by targeting miR-135a-5p. This study suggests that reducing ciRS-7 expression levels or using miR-135a-5p inhibitors could inhibit NP, which could be a potential treatment approach. However, the application of circRNA in NP is still in its initial stage.

#### 4.2.3. Acetylation

In Figure 5, it can be seen that #2 acetylation is the third largest cluster. Histone acetylation is a post-translational modification that relies on two key enzymes: histone deacetylases (HDACs) and histone acetyltransferases (HATs), occurring at the N-terminal end of the protein octamer (Kratz et al., 2010). In recent years, extensive experiments performed on animal models have demonstrated the effectiveness of various HDAC activators and HAT inhibitors in the treatment of NP by targeting specific epigenetic locations. In addition, HDAC and HAT activity and histone acetylation levels are dynamic and can be promising targets for intervention in the treatment of NP. For example, according to some researchers, in type 2 diabetes mellitus (T2DM) model rats, Sirtuin3 (SIRT3), a nicotinamide adenine dinucleotide + (NAD+)-dependent HDAC, deacetylates forkhead box class O3a (FoxO3a) and subsequently inhibits FoxO3a phosphorylation, ubiquitination, and degradation. As a result, SIRT3 stabilizes the FoxO3a protein and prevents oxidative damage, which reduces NP (Khangura et al., 2017; Wang et al., 2018; Zhou et al., 2021). These researchers suggest that SIRT3 activation may serve as a potential therapeutic strategy for NP.

#### 4.2.4. Long non-coding RNA

With more than 200 nucleotides, scholars have found that lncRNAs are key regulators of neuronal function, especially in the NP. Nearly 40% of lncRNAs are found only in the nervous system and are considered important in regulating gene expression (Zhang et al., 2018; Li et al., 2019; Wu et al., 2019; Zhang C. T. et al., 2022). Its biological and molecular mechanisms are diverse and complex, and it has been studied in various NP models, such as sciatic nerve ligation (SNL), CCI, and diabetic NP (DNP) (Hu et al., 2021; Xu et al., 2021). For example, recently, Wen et al. (2021) found that by lowering high mobility group box 1 (HMGB1) expression in a female mouse model of bilateral CCI, lncRNA FIRRE down-regulation reduced the release of microglia-derived pro-inflammatory cytokines, relieving NP in female mice. In conclusion, lncRNA is expected to be a potential regulator for NP.

#### 4.2.5. Microglia

In the aforementioned keyword burst detection, “microglia” has exhibited high burst intensity in recent years. Being the resident immune cells in the central nervous system, microglia cooperate with neurons to maintain homeostasis and detect environmental changes. Its multiple dysregulations are associated with the development and maintenance of NP (Gu et al., 2016; Jeong et al., 2016; Penas and Navarro, 2018; Ji and Xu, 2021). Immune memory of microglia is formed by epigenetically mediated alterations in the target gene enhancer pool (Yeh and Ikezu, 2019). Recently, acetylated histones and their recruitment by bromodomain and extra terminal domain (BET) proteins have been suggested to play a key role in microglia-mediated spinal cord neuroinflammation. And experiments have demonstrated that combining epigenetic inhibitors (HDAC inhibitors) and BET inhibitors to restore abnormal epigenetic modifications is a promising new therapeutic option (Borgonetti and Galeotti, 2021; Borgonetti et al., 2022). Furthermore, Keita Kohno et al. demonstrated that in the behavioral pain hypersensitivity response after peripheral nerve injury, myelin fragments released from A-fiber sensory neurons after the injury can activate CD11c + microglia in the dorsal horn of the spinal cord, which works by secreting insulin-like growth factor-1 (IGF-1) to relieve NP and inhibit pain recurrence (Kohno et al., 2022). This study reveals the mechanisms underlying NP relief and recurrence to provide potential targets for therapeutic strategies, which has dramatically improved our understanding of the epigenetic mechanisms underlying the pathogenesis of NP. In summary, the mechanism of treating NP through microglia has captured the interest of many scholars.

## 5. Strengths and limitations

The information derived from this study was combined for a bibliometric analysis of the directions and trends of epigenetics and NP. However, this study also has several limitations. First, all the documents were downloaded from the SCIE of the WoS Core Collection database. Therefore, the literature included in this study may not represent the whole field of epigenetics and NP. However, this database is known for having strict selection rules and containing high-quality journals and a large number of documents. Moreover, the database is highly compatible with CiteSpace. By contrast, other databases have more restrictions because they analyze other databases on the basis of WoS data format. Notably, it has been noted that the WoS database provides more material in analyzing the past literature and offers greater precision in the categorization of journals in comparison with the Scopus database (Yeung, 2019). Secondly, this study focuses on quantitative analysis, with less qualitative analysis. In addition, non-English papers were not included in this study. Therefore, these factors may have contributed to publication bias.

## 6. Conclusion

Our study uses CiteSpace for visual analysis, and fully reveals the global trend of research on epigenetics and NP by analyzing the number of publications, countries, institutions, authors, keywords, etc. The number of publications is proliferating, and the research is becoming more in-depth and extensive. Tao Yuan-Xiang, Zhang Yi and Wu Shao-Gen are significant researchers who have had a major impact on the study of epigenetics in the NP field. Recent keyword and cluster analysis show that “DNA methylation,” “Circular RNA,” “acetylation,” “long non-coding RNA” and “microglia” may currently be the focus of attention. Out of a total of 867 publications, only 5 clinical studies are found. More rigorous clinical trials and additional studies that explore relevant mechanisms are required in the future.

## Author contributions

JG and BC contributed to the conception, design of the study, and revised the manuscript. CZ, CG, and WZ collected and analyzed the data. CZ and WZ wrote the manuscript. All authors have read and approved the final version of the manuscript.

## Funding

This work was supported by the Natural Science Foundation of Jiangsu Province (grant no. BK20210907), Medical Research Project of Jiangsu Commission of Health (grant. no. Z2022004), and Research Foundation for Talented Scholars of Xuzhou Medical University (grant no. D2020056).

## Conflict of interest

The authors declare that the research was conducted in the absence of any commercial or financial relationships that could be construed as a potential conflict of interest.

## Publisher’s note

All claims expressed in this article are solely those of the authors and do not necessarily represent those of their affiliated organizations, or those of the publisher, the editors and the reviewers. Any product that may be evaluated in this article, or claim that may be made by its manufacturer, is not guaranteed or endorsed by the publisher.

## References

[ref1] Bird A. Taggart M. Frommer M. Miller O. J. Macleod D. (1985). A fraction of the mouse genome that is derived from islands of nonmethylated. CpG-rich DNA. Cell 40, 91–99. doi: 10.1016/0092-8674(85)90312-5, PMID: 2981636

[ref2] Borgonetti V. Galeotti N. (2021). Combined inhibition of histone deacetylases and BET family proteins as epigenetic therapy for nerve injury-induced neuropathic pain. Pharmacol. Res. 165:105431. doi: 10.1016/j.phrs.2021.105431, PMID: 33529752

[ref3] Borgonetti V. Meacci E. Pierucci F. Romanelli M. N. Galeotti N. (2022). Dual HDAC/BRD4 inhibitors relieves neuropathic pain by attenuating inflammatory response in microglia after spared nerve injury. Neurotherapeutics 19, 1634–1648. doi: 10.1007/s13311-022-01243-6, PMID: 35501470 PMC9606187

[ref4] Cai W. Zhang Y. Su Z. (2020). ciRS-7 targeting miR-135a-5p promotes neuropathic pain in CCI rats via inflammation and autophagy. Gene 736:144386. doi: 10.1016/j.gene.2020.144386, PMID: 31978512

[ref5] Cavalli G. Heard E. (2019). Advances in epigenetics link genetics to the environment and disease. Nature 571, 489–499. doi: 10.1038/s41586-019-1411-0, PMID: 31341302

[ref6] Chappell G. Pogribny I. P. Guyton K. Z. Rusyn I. (2016). Epigenetic alterations induced by genotoxic occupational and environmental human chemical carcinogens: a systematic literature review. Mutat Res Rev Mutat Res 768, 27–45. doi: 10.1016/j.mrrev.2016.03.004, PMID: 27234561 PMC4884606

[ref7] Chen C. M. (2006). CiteSpace II: detecting and visualizing emerging trends and transient patterns in scientific literature. J. Am. Soc. Inf. Sci. Technol. 57, 359–377. doi: 10.1002/asi.20317

[ref8] Cheng X. D. Roberts R. J. (2001). AdoMet-dependent methylation, DNA methyltransferases and base flipping. Nucleic Acids Res. 29, 3784–3795. doi: 10.1093/nar/29.18.3784, PMID: 11557810 PMC55914

[ref9] Ferioli M. Zauli G. Maiorano P. Milani D. Mirandola P. Neri L. M. (2019). Role of physical exercise in the regulation of epigenetic mechanisms in inflammation, cancer, neurodegenerative diseases, and aging process. J. Cell. Physiol. 234, 14852–14864. doi: 10.1002/jcp.28304, PMID: 30767204

[ref10] Fernandez-de-las-Penas C. Nijs J. Neblett R. Polli A. Moens M. Goudman L.. (2022). Phenotyping post-COVID pain as a nociceptive, neuropathic, or Nociplastic pain condition. Biomedicine 10:2562. doi: 10.3390/biomedicines10102562, PMID: 36289827 PMC9599440

[ref11] Frances R. Mata-Garrido J. de la Fuente R. Carcelen M. Lafarga M. Berciano M. T.. (2022). Identification of epigenetic interactions between MicroRNA-30c-5p and DNA methyltransferases in neuropathic pain. Int. J. Mol. Sci. 23:13994. doi: 10.3390/ijms232213994, PMID: 36430472 PMC9694031

[ref12] Franco-Enzastiga U. Garcia G. Murbartian J. Gonzalez-Barrios R. Salinas-Abarca A. B. Sanchez-Hernandez B.. (2021). Sex-dependent pronociceptive role of spinal alpha(5)-GABA(a) receptor and its epigenetic regulation in neuropathic rodents. J. Neurochem. 156, 897–916. doi: 10.1111/jnc.15140, PMID: 32750173

[ref13] Gu N. Eyo U. B. Murugan M. Peng J. Y. Matta S. Dong H. L.. (2016). Microglial P2Y12 receptors regulate microglial activation and surveillance during neuropathic pain. Brain Behav. Immun. 55, 82–92. doi: 10.1016/j.bbi.2015.11.007, PMID: 26576724 PMC4864135

[ref14] Hicks D. Wouters P. Waltman L. de Rijcke S. Rafols I. (2015). Bibliometrics: the Leiden manifesto for research metrics. Nature 520, 429–431. doi: 10.1038/520429a, PMID: 25903611

[ref15] Holliday R. (2005). DNA methylation and epigenotypes. Biochem.-Moscow 70, 500–504. doi: 10.1007/s10541-005-0144-x, PMID: 15948704

[ref16] Hu C. He M. L. Xu Q. Tian W. Q. (2021). Advances with non-coding RNAs in neuropathic pain. Front. Neurosci. 15:760936. doi: 10.3389/fnins.2021.760936, PMID: 35002601 PMC8733285

[ref17] Huang Y. Li X. Tao G. Zhu T. Lin J. (2017). Comparing serum microRNA levels of acute herpes zoster patients with those of postherpetic neuralgia patients. Medicine 96:e5997. doi: 10.1097/MD.0000000000005997, PMID: 28225487 PMC5569417

[ref18] Jensen T. S. Baron R. Haanpaa M. Kalso E. Loeser J. D. Rice A. S. C.. (2011). A new definition of neuropathic pain. Pain 152, 2204–2205. doi: 10.1016/j.pain.2011.06.017, PMID: 21764514

[ref19] Jeong H. Na Y. J. Lee K. Kim Y. H. Lee Y. Kang M.. (2016). High-resolution transcriptome analysis reveals neuropathic pain gene-expression signatures in spinal microglia after nerve injury. Pain 157, 964–976. doi: 10.1097/j.pain.0000000000000470, PMID: 26761385

[ref20] Ji A. J. Xu J. B. (2021). Neuropathic pain: biomolecular intervention and imaging via targeting microglia activation. Biomol. Ther. 11:1343. doi: 10.3390/biom11091343, PMID: 34572554 PMC8466763

[ref21] Jiang W. Tan X. Y. Li J. M. Yu P. Dong M. (2022). DNA methylation: a target in neuropathic pain. Front. Med. 9:879902. doi: 10.3389/fmed.2022.879902, PMID: 35872752 PMC9301322

[ref22] Khangura R. K. Bali A. Jaggi A. S. Singh N. (2017). Histone acetylation and histone deacetylation in neuropathic pain: an unresolved puzzle? Eur. J. Pharmacol. 795, 36–42. doi: 10.1016/j.ejphar.2016.12.001, PMID: 27916557

[ref23] Kleinberg J. (2002). Bursty and hierarchical structure in streams, In Proceedings of the eighth ACM SIGKDD international conference on knowledge discovery and data mining. (Edmonton, Alberta, Canada: Association for Computing Machinery).

[ref24] Kohno K. Shirasaka R. Yoshihara K. Mikuriya S. Tanaka K. Takanami K.. (2022). A spinal microglia population involved in remitting and relapsing neuropathic pain. Science 376:6588. doi: 10.1126/science.abf6805, PMID: 35357926

[ref25] Kratz A. Arner E. Saito R. Kubosaki A. Kawai J. Suzuki H.. (2010). Core promoter structure and genomic context reflect histone 3 lysine 9 acetylation patterns. BMC Genomics 11:257. doi: 10.1186/1471-2164-11-257, PMID: 20409305 PMC2867832

[ref26] Li Z. Li X. Y. Chen X. Li S. G. Ho I. H. T. Liu X. D.. (2019). Emerging roles of long non-coding RNAs in neuropathic pain. Cell Prolif. 52:e12528. doi: 10.1111/cpr.12528, PMID: 30362191 PMC6430490

[ref27] Li X. Wang D. Zhou J. Yan Y. Chen L. (2020). Evaluation of circulating microRNA expression in patients with trigeminal neuralgia an observational study. Medicine 99:e22972. doi: 10.1097/MD.0000000000022972, PMID: 33235064 PMC7710236

[ref28] Mao S. S. Zhang S. S. Zhou S. S. Huang T. Feng W. Gu X. S.. (2019). A Schwann cell-enriched circular RNA circ-Ankib1 regulates Schwann cell proliferation following peripheral nerve injury. FASEB J. 33, 12409–12424. doi: 10.1096/fj.201900965R, PMID: 31415184 PMC6902728

[ref29] Mathias L. Nurcan U. Anna T. Claudia S. (2017). Aberrant microRNA expression in patients with painful peripheral neuropathies. J. Neurol. Sci. 380, 242–249. doi: 10.1016/j.jns.2017.07.041, PMID: 28870579

[ref30] Memczak S. Jens M. Elefsinioti A. Torti F. Krueger J. Rybak A.. (2013). Circular RNAs are a large class of animal RNAs with regulatory potency. Nature 495, 333–338. doi: 10.1038/nature11928, PMID: 23446348

[ref31] Pan Z. Q. Li G. F. Sun M. L. Xie L. Liu D. Zhang Q.. (2019). MicroRNA-1224 splicing CircularRNA-Filip1l in an Ago2-dependent manner regulates chronic inflammatory pain via targeting Ubr5. J. Neurosci. 39, 2125–2143. doi: 10.1523/JNEUROSCI.1631-18.2018, PMID: 30651325 PMC6507086

[ref32] Park C. Rosenblat J. D. Brietzke E. Pan Z. H. Lee Y. Cao B.. (2019). Stress, epigenetics and depression: a systematic review. Neurosci. Biobehav. Rev. 102, 139–152. doi: 10.1016/j.neubiorev.2019.04.010, PMID: 31005627

[ref33] Penas C. Navarro X. (2018). Epigenetic modifications associated to neuroinflammation and neuropathic pain after neural trauma. Front. Cell. Neurosci. 12:158. doi: 10.3389/fncel.2018.00158, PMID: 29930500 PMC5999732

[ref34] Polli A. Ickmans K. Godderis L. Nijs J. (2019). When environment meets genetics: a clinical review of the epigenetics of pain, psychological factors, and physical activity. Arch. Phys. Med. Rehabil. 100, 1153–1161. doi: 10.1016/j.apmr.2018.09.118, PMID: 30352223

[ref35] Portela A. Esteller M. (2010). Epigenetic modifications and human disease. Nat. Biotechnol. 28, 1057–1068. doi: 10.1038/nbt.1685, PMID: 20944598

[ref36] Sanger H. L. Klotz G. Riesner D. Gross H. J. Kleinschmidt A. K. (1976). Viroids are single-stranded covalently closed circular RNA molecules existing as highly base-paired rod-like structures. Proc. Natl. Acad. Sci. U. S. A. 73, 3852–3856. doi: 10.1073/pnas.73.11.3852, PMID: 1069269 PMC431239

[ref37] Stenz L. Le Carre J. Luthi F. Vuistiner P. Burrus C. Paoloni-Giacobino A.. (2022). Genome-wide epigenomic analyses in patients with nociceptive and neuropathic chronic pain subtypes reveals alterations in methylation of genes involved in the neuro-musculoskeletal system. J. Pain 23, 326–336. doi: 10.1016/j.jpain.2021.09.001, PMID: 34547430

[ref38] Sun Z. W. Waybright J. M. Beldar S. Chen L. Foley C. A. Norris-Drouin J. L.. (2022). Cdyl deficiency brakes neuronal excitability and nociception through promoting Kcnb1 transcription in peripheral sensory neurons. Adv. Sci. 9:e2104317–e2104317. doi: 10.1002/advs.202104317, PMID: 35119221 PMC8981457

[ref39] Sun N. Yu L. A. Gao Y. B. Ma L. F. Ren J. X. Liu Y.. (2021). MeCP2 epigenetic silencing of *Oprm1* gene in primary sensory neurons under neuropathic pain conditions. Front. Neurosci. 15:743207. doi: 10.3389/fnins.2021.743207, PMID: 34803588 PMC8602696

[ref40] Wang X. Shen X. Xu Y. Xu S. Xia F. Zhu B.. (2018). The etiological changes of acetylation in peripheral nerve injury-induced neuropathic hypersensitivity. Mol. Pain 14:1744806918798408. doi: 10.1177/1744806918798408, PMID: 30105933 PMC6144590

[ref41] Wen Y. Fan X. Bu H. Ma L. Kong C. Huang C.. (2021). Downregulation of lncRNA FIRRE relieved the neuropathic pain of female mice by suppressing HMGB1 expression. Mol. Cell. Biochem. 476, 841–852. doi: 10.1007/s11010-020-03949-7, PMID: 33151463

[ref42] Wu W. Ji X. J. Zhao Y. (2019). Emerging roles of long non-coding RNAs in chronic neuropathic pain. Front. Neurosci. 13:1097. doi: 10.3389/fnins.2019.01097, PMID: 31680832 PMC6813851

[ref43] Xu S. C. Dong H. Zhao Y. Feng W. (2021). Differential expression of long non-coding RNAs and their role in rodent neuropathic pain models. J. Pain Res. 14, 3935–3950. doi: 10.2147/jpr.S344339, PMID: 35002313 PMC8722684

[ref44] Yeh H. Ikezu T. (2019). Transcriptional and epigenetic regulation of microglia in health and disease. Trends Mol. Med. 25, 96–111. doi: 10.1016/j.molmed.2018.11.004, PMID: 30578089 PMC6377292

[ref45] Yeung A. W. K. (2019). Comparison between Scopus, web of science, PubMed and publishers for mislabelled review papers. Curr. Sci. 116:1909. doi: 10.18520/cs/v116/i11/1909-1914

[ref46] Yin X. M. Zheng W. He L. Mu S. K. Shen Y. L. Wang J. X. (2022). CircHIPK3 alleviates inflammatory response and neuronal apoptosis via regulating miR-382-5p/DUSP1 axis in spinal cord injury. Transpl. Immunol. 73:101612. doi: 10.1016/j.trim.2022.101612, PMID: 35500847

[ref47] Zhang C. T. Gao R. Zhou R. H. Chen H. Liu C. L. Zhu T.. (2022). The emerging power and promise of non-coding RNAs in chronic pain. Front. Mol. Neurosci. 15:1037929. doi: 10.3389/fnmol.2022.1037929, PMID: 36407760 PMC9668864

[ref48] Zhang J. Chen S.-R. Zhou M.-H. Jin D. Chen H. Wang L.. (2022). HDAC2 in primary sensory neurons constitutively restrains chronic pain by repressing alpha2delta-1 expression and associated NMDA receptor activity. J. Neurosci. 42, 8918–8935. doi: 10.1523/jneurosci.0735-22.2022, PMID: 36257688 PMC9732832

[ref49] Zhang Z. Tao W. J. Hou Y. Y. Wang W. Kenny P. J. Pan Z. Z. Z. (2014). MeCP2 repression of G9a in regulation of pain and morphine reward. J. Neurosci. 34, 9076–9087. doi: 10.1523/JNEUROSCI.4194-13.2014, PMID: 24990928 PMC4078085

[ref50] Zhang Y. W. Tao Y. Liao Q. (2018). Long noncoding RNA: a crosslink in biological regulatory network. Brief. Bioinform. 19, 930–945. doi: 10.1093/bib/bbx042, PMID: 28449042

[ref51] Zhang H. H. Zhang Y. L. Wang X. X. Yang P. P. Zhang B. Y. Hu S. F.. (2020). Circular RNA profile in diabetic peripheral neuropathy: analysis of coexpression networks of circular RNAs and mRNAs. Epigenomics 12, 843–857. doi: 10.2217/epi-2020-0011, PMID: 32212929

[ref52] Zhao X. L. Tang Z. X. Zhang H. K. Atianjoh F. E. Zhao J. Y. Liang L. L.. (2013). A long noncoding RNA contributes to neuropathic pain by silencing Kcna2 in primary afferent neurons. Nat. Neurosci. 16, 1024–1031. doi: 10.1038/nn.3438, PMID: 23792947 PMC3742386

[ref53] Zheng J. Z. Zhou R. Meng B. Y. Li F. R. Liu H. M. Wu X. B. (2021). Knowledge framework and emerging trends in intracranial aneurysm magnetic resonance angiography: a scientometric analysis from 2004 to 2020. Quant. Imaging Med. Surg. 11, 1854–1869. doi: 10.21037/qims-20-729, PMID: 33936970 PMC8047370

[ref54] Zhou Z. B. Niu Y. L. Huang G. X. Lu J. J. Chen A. M. Zhu L. (2018). Silencing of circRNA.2837 plays a protective role in sciatic nerve injury by sponging the miR-34 family via regulating neuronal autophagy. Mol. Ther. Nucl. Acids 12, 718–729. doi: 10.1016/j.omtn.2018.07.011, PMID: 30098504 PMC6088565

[ref55] Zhou C. H. Zhang Y. F. Jiao X. W. Wang G. Z. Wang R. Y. Wu Y. Q. (2021). YSIRT3 alleviates neuropathic pain by deacetylating FoxO3a in the spinal dorsal horn of diabetic model rats. Reg. Anesth. Pain Med. 46, 49–56. doi: 10.1136/rapm-2020-101918, PMID: 33127810

